# Reliability and Validity of the Arabic Version of a Questionnaire Assessing Pain, Discomfort and Related Jaw Function Impairment after Extraction of Primary Teeth in Children

**DOI:** 10.3390/dj8040120

**Published:** 2020-10-19

**Authors:** Reem Naaman, Azza A. El-Housseiny, Najlaa Alamoudi, Narmin Helal, Rahaf Sahhaf

**Affiliations:** 1 Pediatric Dentistry Department, Faculty of Dentistry, King Abdulaziz University, Jeddah 21589, Saudi Arabia; dr.reem.naaman@hotmail.com (R.N.); or or azza.elhousseiny@dent.alex.edu.eg (A.A.E.-H.); nhilal@kau.edu.sa (N.H.); dr.rsahhaf@gmail.com (R.S.); 2 Pediatric Dentistry Department, Faculty of Dentistry, Alexandria University, Alexandria 21526, Egypt

**Keywords:** children, pain, dental extraction, validity, reliability

## Abstract

This study aims to translate a previously published English language questionnaire that assessed pain and discomfort after the extraction of primary teeth in children into Arabic, and evaluate its validity and reliability. All participating children (*n* = 120), aged 9 to 12-years-old, completed the 33-item Arabic version questionnaire after the extraction procedure had taken place. The questionnaire included three parts that were completed at three different times, namely, immediately, the first evening, and one week after the extraction procedure. Internal consistency, content validity, criterion validity, and factor analysis were performed. The results showed a good internal consistency (Cronbach’s alpha = 0.83), acceptable criterion validity with a significantly strong correlation with the Visual Analog Scale (VAS), and satisfactory content validity (average content validity index (CVI = 0.90). The final factor model was comprised of four factors with an eigenvalue greater than 1, explaining 70% of the common variance. The identified factors were labeled as follows: Factor 1—analgesic consumption; Factor 2—expression of discomfort from the extraction site; Factor 3—perception of masticatory capability; and Factor 4—pain/discomfort from the dental extraction procedure. Based on the results, a shorter form of the questionnaire had satisfactory psychometric characteristics and can be used with children within the selected age group.

## 1. Introduction

The International Association for the Study of Pain (IASP) defines pain as an unpleasant emotional experience associated with actual or relative tissue damage [1]. The IASP also described a painful experience as being subjective, i.e., the response differs from one individual to another. This could be related to age, cognitive awareness, the ability to cope with different situations, or the person’s developmental stage.

The most important factor in shaping a person’s pain response is their past experiences, particularly those in early life. As pediatric dentists in clinical settings, it is very important to minimize painful experiences during dental procedures on children. Traumatic experiences will most likely play a significant role in shaping children’s pain response in the future and result in increased distress at future dental visits if not managed properly [2].

Dental extraction is one of the most common procedures that may cause unpleasant experiences, particularly in children, and to be successful, it must be efficient and cause minimal pain and discomfort.

Pain assessment is the first step in adequate pain management. Appropriate pain assessment will enable the dentist to select the proper pain control strategies, to use a suitable behavior management technique to ensure a pain-free dental treatment [3]. Self-reporting is considered the gold standard tool for pain assessment in pediatric patients. It helps pediatric dentists to recognize the degree and intensity of pain or discomfort that pediatric patients experience during dental-extraction procedures. Studies have found that pain assessment by healthcare providers was not considered reliable and tended to underestimate the self-reporting scores from children [4].

The main indicators of any questionnaire’s quality level are its validity and reliability [5]. The basic concept behind reliability is simply the ability to measure something in a reproducible manner, while validity relates to whether the tool is measuring what it is supposed to measure [6]. Validating a questionnaire largely reduces errors and improves the accuracy of the measurement process. It also helps to analyze whether the questionnaire is well-developed and easily completed by the selected age group. There are several scales available in the literature to assess pain in children, such as the Face, Legs, Arms, Cry, Consolability (FLACC) scale [7], the Visual Analog Scale (VAS) [8], the Wong-Baker FACES Pain Rating Scale (WBS) [9], and the Colored Analog Scale (CAS) [10]. However, these are general tools and are not focused on the extraction related pain behaviors that children can show. Dental extraction is a procedure that involves different levels of pain or discomfort experienced from local anesthesia, the extraction procedure itself, and the feeling after the procedure has been completed. It is important to develop a tool that identifies the pain of an extraction procedure at different time occasions with all the pain indicators being well addressed. For example, a full description of the pain, pain medications, and the impact of pain on the child’s functioning should all be involved. To our knowledge, only one English-language study was found in the literature that used a questionnaire as an assessment tool to report pain experiences after dental extraction in children [11]. The questionnaire that this study used was adopted from two previously published studies [12,13] that both showed acceptable reliability and validity when the English-language questionnaire was tested. However, one study assessed pain from orthodontic treatment in adolescents [13], and the other assessed the functioning of temporomandibular joints for patients with temporomandibular disorders (TMD) in adults [12]. It is, thus, necessary to investigate the questionnaire in a children’s context. Moreover, children’s pain perception may differ due to interethnic differences based on various sociocultural factors [2,14]. Considering that no Arabic-language version of a questionnaire is available to assess pain after extraction procedures in Arabic-speaking children, this study aims to translate the questionnaire into the Arabic language and then assess its reliability and validity.

## 2. Materials and Methods 

One hundred-and-twenty pediatric patients participated in this study. It was a cross-sectional non-randomized study design. Recruitment of patients was conducted at the University Dental Hospital at King Abdulaziz University in Jeddah, Saudi Arabia, over 28 months between September 2017 and December 2019. The inclusion criteria included the following: Children aged 9 to 12; the presence of firm primary teeth with roots exhibiting minimal or no resorption radiographically indicated for extraction; selected primary teeth were planned for extraction due to extensive carious lesions and a lack of restorability or for interceptive treatment reasons; absence of clinical signs of extensive infection, such as extra-oral swelling that needs the prescription of antibiotics prior to extraction; the children must be able to speak, read, and understand the Arabic language. Children with any systemic conditions or mental disabilities were excluded. All subjects completed the self-report questionnaire after the dental-extraction procedures. The operator explained the questionnaire to each child before performing the dental-extraction procedure. A signed consent for each child’s participation and video recording was obtained from at least one parent, and ethical approval was obtained from the Research Ethics Committee of the Faculty of Dentistry at King Abdulaziz University (No. 073-09-17).

### 2.1. Sample Size Calculation

A convenience sample of 120 participants was chosen as the minimum required number to run internal consistency and factor analysis tests [15].

### 2.2. Questionnaire Description 

The Arabic version of the questionnaire was adapted from the English version, published by Naoumova et al. [11]. As mentioned earlier, two previously used questionnaires were combined into one questionnaire that had originally been developed by Feldmann et al. [13] and Stagenga et al. [12]. Both studies tested its reliability and validity and found them to be acceptable.

**Figure 1:**
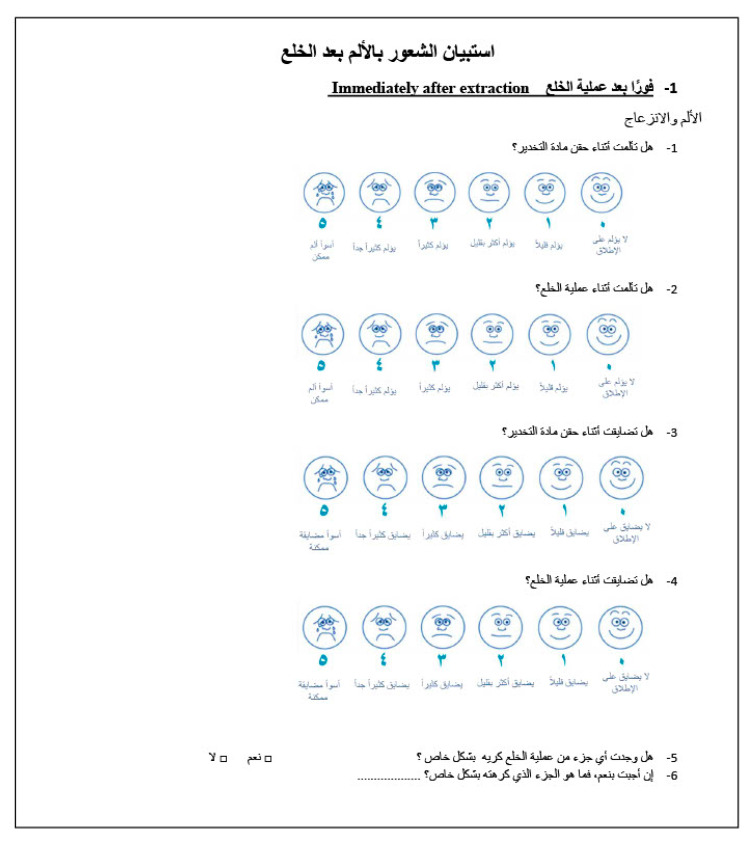
A sample of the first part of the Arabic post-extraction pain experience questionnaire. The first 4 questions were on pain and discomfort from local anesthesia and extraction and were graded on a modified Wong-Baker Faces Pain Rating Scale.

The Arabic version of the questionnaire was comprised of 33 items and was divided into four parts. Each child filled out Part I, “6 items,” immediately after the extraction procedure had been performed. Questions 1–4 concerned pain and discomfort experienced from local anesthesia and extraction procedures. These questions were graded on a modified Wong-Baker Faces Pain Rating Scale [9] ranging from 0 to 5, with “no pain at all” to “worst pain possible” or “no discomfort” to “worst discomfort possible” as the extremes. Children were asked to point to the face that best described their pain or discomfort. Question 5 had a binary response (yes/no) as follows: “Did you experience any part of the extraction as particularly unpleasant?” Question 6 was an open question to allow comment on Question 5 if the answer was yes (Figure 1). Part II, “4 items,” was completed via a phone interview with the child on the first evening after the extraction procedure. If the child was not available to take the call, he or she was contacted at a later time. Questions 1 and 2 in this part were about pain and discomfort from the extraction site, rated on a five-point scale similar to the one in the previous part. Questions 3 and 4 concerned analgesic consumption. Part III, “13 items,” was completed one week after the extraction and was divided into three sections. The first, “2 items” concerned pain and discomfort from the extraction site after one week. The second section, “6 items,” concerned analgesic consumption during the week after the extraction procedure. Each question in this part had yes/no responses, with open-ended questions depending on the answers. The third section, “5 items,” concerned changes in daily activities during the week after the dental extraction. Three questions asked for binary responses (yes/no), and two questions were open-ended. Part IV also was completed after one week and included a total of 10 items related to jaw function, with four items related to eating specific food types, two items related to psychosocial activities, and four items related to mandibular function. Each item was rated as follows: 0 = no difficulties; 1 = some difficulties; 2 = very difficult; 3 = extremely difficult (Table 1).

Four items were repeated measurements collected at two-time points, namely, from the first evening after extraction and from the first week after extraction. These items were: Pain from extraction site, discomfort from extraction site, analgesic consumption, and kind of analgesics. These items were marked with an asterisk * in Table 1.

#### 2.2.1. Forward Translation

The questionnaire was translated into Arabic according to the stages of the cross-cultural adaptation process of self-report measures recommended by Beaton et al. [16]. Two bilingual translators conducted the translation independently. Both translators’ native language was Arabic, and each translator presented a written report of the translated questionnaire. Both translators set up a committee with a third evaluator, who was a professor from the medical field, to compare the translated Arabic versions and then agree on the first translated Arabic version.

#### 2.2.2. Backward Translation

Two other translators, who were not from the medical field and who had not seen the original English version of the questionnaire, translated the first Arabic version of the questionnaire back into English to ensure that the translated version matched the original. Both translators were native Arabic speakers, but both understood and spoke English fluently, and one was a certified English language teaching consultant. A committee then reviewed and compared both translated reports with the original English version for any discrepancies. Minor adjustments were then made, with some terms being replaced with simpler ones to make it easier for the children to understand. For example, “resistance when chewing” was replaced with the term “chewing on the extraction site”. The reviewers came to a consensus and produced the pre-final Arabic version of the questionnaire.

### 2.3. Pilot Study

The pre-final version of the questionnaire was pre-tested on a group of 10 separate children aged 9 to 12, who were not part of the main study sample. The purpose of this pre-testing was to determine the questions’ clarity, i.e., how well children aged 9 to 12 understood the questions [17]. A table was added at the end of the questionnaire asking the children about their thoughts and experiences regarding the questions. All items in the questionnaire were added to the table, and the children were asked to say whether or not each question was clear and to recommend any changes for any item on the questionnaire. Minor adjustments to content and format were made following the feedback received. Only then did the committee formulate the final Arabic version of the questionnaire (Table 1).

### 2.4. Reliability Tests

Internal consistency, a commonly used measure of reliability, was used in this study. It investigated whether all the questionnaire items were homogeneous and highly intercorrelated. Cronbach’s alpha coefficient was used to assess internal consistency. The generally accepted minimum value was 0.7 [18,19].

### 2.5. Validity Tests 

Content validity concerns the correspondence level for a group of items, representing the construct of interest [20]. The assessment of content validity starts at the earliest point of instrument development [21]. This can be done through a two-stage process as described by Lynn et al., namely, development and judgment quantification [22]. After the instrument had been constructed, five experts were invited to participate in evaluating the adequacy of the instrument to be used in this study. Three of the experts were from the Pediatric Dentistry Department, and two were from the Oral and Maxillofacial Surgery Department. Each expert was given a booklet containing a cover letter and a copy of the questionnaire with a content validity assessment form. The cover letter included the title of the study, the main objectives, and the reason for choosing him/her as a content expert. Experts were asked to rate each item on a four-point scale, based on the following dimensions: relevance, clarity, simplicity, and ambiguity. The experts were asked to provide reasons why they had given a score of less than 4 for any item on the scale. They were also requested to mention if they thought the item required revision or even deletion.

The content validity index for each item (I-CVI) was computed based on the experts’ ratings. The I-CVI was calculated by the proportion of experts who gave a rating of 3 or 4, divided by the total number of experts. I-CVIs were compared with modified kappa values that were developed by Polit et al. to adjust each I-CVI value for a chance agreement [23]. Items with I-CVIs greater than 0.78 were deemed excellent. Any item with an I-CVI of less than 0.78 was a candidate for revision or deletion. The instrument level-CVI or Average-CVI was also calculated by summing all the items’ CVIs and then dividing it by the number of items. An average of 0.90 or higher is recommended [21]. Finally, the average of the proportion of items that were rated relevant by the experts was calculated.

Criterion validity depended on how well the scores, obtained through the questionnaire, correlated with the scores obtained from other valid scales, preferably, a “gold standard”, which has been approved in the field [5,6]. In our study, we used the visual analog scale (VAS) [8], as well as the sound, eye, motor scale (SEM) [24].

Each child’s pain reaction during local anesthesia administration and dental extraction procedures was videotaped. The recordings were evaluated by a trained calibrated pediatric dentist to complete the sound, eye, and motor scale (SEM) (intra-rater reliability; percent agreement 0.92; kappa = 0.76). The ratings incorporated three types of observations (sound, eye, and motor) with both local anesthesia and extraction procedures. The response level was given a numerical value from 1 to 4, with 1 indicating comfort, 2 indicating mild discomfort, 3 indicating moderate pain, and 4 indicating a high level of pain (Table 2).

To assess pain perception, the visual analog scale (VAS) was also used by the children immediately after the extraction procedure. The scale is a 100 mm straight line ranging from “no pain” at one end to “worst pain imaginable” at the other end [25]. It was used for pain assessment of both local anesthesia and extraction procedures. The rating of both scales was then compared with the first part of the questionnaire scores. Spearman’s rho correlation coefficient was employed to assess the correlation between the SEM and VAS scores, as well as the pain-experience questionnaire answers.

Factor Analysis (Principal Component Analysis (PCA)) Exploratory factor analysis (principal component analysis) was used in this study to determine the factor structure Arabic-version of the questionnaire. This is a well-known technique to identify the underlying structure among the scale’s variables [15]. Items that are highly correlated will share the same so-called factor.

An eigenvalue represents the variance level explained by each factor. Eigenvalues above 1.00 are deemed strong enough to be retained. Eigenvalues indicate the maximum number of factors that can be extracted for the factor matrix. After extraction of the factors, a rotation process was performed. Such a rotation was employed to obtain a more simplified and meaningful factor structure. The most commonly used rotation techniques are orthogonal and oblique factor rotations. In our study, we chose the orthogonal factor rotation method (Varimax).

Each variable (item) influences a factor, and this is called factor loading. The factor-loading level demonstrates how well the variable is correlated to a factor. Factor loadings from 0.30 to 0.40 are viewed as having the minimum acceptable levels for factor matrix interpretation. Based on our sample size, a factor loading of 0.5 or greater is required to achieve a significant interpretation [15]. The Kaiser-Meyer-Olkin (KMO) measure was calculated to determine whether the sample was adequate for factor analysis.

All data were analyzed using SAS software (Version 9.4; SAS Institute Inc.; Cary, NC, USA).

## 3. Results

One hundred-and-twenty healthy pediatric patients were included in this study, 66 females (55%) and 54 males (45%). The mean age was 9.9 years old and ranged from 9-to-12 years old. Forty-two children had primary teeth extractions for orthodontic reasons (35%), and seventy-eight had extractions of non-restorable carious primary teeth (65%). The questionnaire was not completed by one child due to loss of contact, another questionnaire had one part missing, and the VAS rating was not completed by another child. These were replaced by three other children.

### 3.1. Internal Consistency 

The total Cronbach Coefficient alpha was 0.83, above 0.8, indicating excellent reliability of the Arabic version of the pain experience questionnaire. The sensitivity analysis of using the four repeated items from the first evening after extraction yielded exactly the same total Cronbach Coefficient alpha of 0.83, indicating excellent reliability of the Arabic version of the pain experience questionnaire, regardless of the different time-points at which those four items were collected.

### 3.2. Criterion Validity 

The results are presented in (Table 3). The VAS scale rating showed a strong correlation with the first part of the questionnaire rating of pain r = 0.75 (*p* < 0.0001). However, a weak correlation was found between the first part of the questionnaire ratings and the SEM scale scores r = 0.36 (*p* = 0.01).

### 3.3. Content Validity 

A content validity index for each item (I-CVI) was computed based on the ratings of the experts. The I-CVI was calculated as the number of experts giving a rating of 3 or 4, divided by the total number of experts. All the items had I-CVI above 0.78, indicating excellent agreement among the experts that items were relevant. Only one item had an index score of 0.6 (item 1.5) and was a candidate for deletion. The instrument level-CVI was 0.90. The average proportion of agreement across the experts was also 0.90. The results are presented in Table 4.

### 3.4. Factor Analysis 

The main factor analysis started on 27 items, including four items collected immediately after extraction and 23 other items collected one week after extraction. The main analysis included the four repeated items collected from the first week after extraction. Sensitivity analyses were then performed to include the four repeated items, collected from the first evening after extraction, to assess whether the findings of the two models were consistent. Children’s responses to items were checked prior to EFA being performed. Items 3.7 (use of prescription drugs) and 3.8 (kind of analgesic) were removed because all answers were “no” or “none” without any variability. Items 3.9 (school absence) and 3.11 (refraining from leisure activities) were removed as they both had a perfect correlation with items 3.10 (number of school absence days) and 3.12 (kind of activities refrained), respectively. This resulted in 23 remaining items for the EFA analysis.

The Kaiser-Meyer-Olkin (KMO) measure value was 0.71, indicating that the sample was adequate for factor analysis. There were eight factors with eigenvalues above 1.00. The scree plot showed an elbow drop at the 4th factor; hence, we ran the analysis to extract four factors only. The Varimax orthogonal rotation technique was chosen; the rotated factor pattern is presented in Table 5.

A few more steps were taken before reaching the final model. First, item 3.10 (number of school absences) had communality values <0.20 and was removed, leaving 22 items for analysis. Second, 6 items with factor loading <0.5 were removed sequentially; these were: 3.1 (pain from the extraction site after 1 week); 3.12 (kind of activities refrained); 3.13 (sleep disturbance); 4.7 (to drink); 4.8 (to laugh); and 4.10 (to yawn). Finally, item 4.3 (to take a big bite) was removed due to cross-loading between factor 2 and factor 3. 

The final factor matrix exhibited 15 items, which accounted for 70% of the common variance. The Kaiser-Meyer-Olkin (KMO) measure value was improved from 0.71 to 0.75. Factor 1 explained 29.2% of the common variance, factor 2 explained 17.5% of the common variance, factor 3 explained 12.7%, and factor 4 explained 10.3% of the common variance. Factors were labeled as follows: factor 1 (4 items) analgesic consumption, factor 2 (4 items) expression of discomfort from the extraction site, Factor 3 (3 items) perception of masticatory ability and factor 4 (4 items) pain/discomfort from the dental extraction procedure. Factor loadings from the sensitivity analyses (Not shown to conserve space) by using the four items from the first evening (Items 2.1, 2.2, 2.3, and 2.4) were consistent with those, presented in Table 5, for using the four items from the first week after extraction (Items 3.1, 3.2, 3.3, and 3.6).

## 4. Discussion

This study evaluated the reliability and validity of the Arabic version of a questionnaire, originally in the English language, that had been used previously by Naoumova et al. [11] to assess pain and discomfort after the extraction of teeth in children. It was necessary to reassess the instrument’s psychometric properties for using it across different age and cultural contexts.

Regarding sample-size calculation, although sample-size is significant when running reliability and validity tests, particularly for internal consistency and factor analysis tests, the literature contains varying opinions on this particular issue. One study mentioned that a sample-size of 100 participants or greater is preferable [15], while another called for five times the number of items [26]. Another common recommendation was to use the sample-to-variable ratio N:*p*, in which N refers to the number of participants and *p* to the number of variables [27]. A researcher should have a subjects-to-variables ratio of 4:1 or higher [28]. Another study suggested that a more acceptable ratio is 10:1 or greater [15]. Finally, two studies mentioned that these rules are misleading and that less emphasis should be placed on sample-size. Extra caution should be made with variable selection, high communality values, and over-determination of factors [28,29]. Therefore, a sample of 120 participants was within the acceptable range. 

Few modifications were made to the English version, used by Naoumova et al. [11], to make it easier for children to understand and complete. Therefore, we asked the questions about pain and discomfort during local anesthesia administration or extraction procedures immediately following the procedure instead of asking these questions later in the evening, as proposed by Naoumova et al. [11]. With this approach, the child would clearly remember the feeling of pain or discomfort. For example, the sensation of numbness or the unpleasant taste from local anesthesia, which mainly contribute to discomfort, would still be present. This left only two questions about pain and discomfort from the extraction site to be asked on the first evening to avoid confusing the child.

Next, the modified Wong-Baker Faces Pain Scale was used instead of the Visual Analog Scale (VAS), which allowed children to point at the face that best described their pain. This was an easier method for pain assessment in children, compared to the use of the VAS (Figure 1). This is in agreement with a study that found a significant preference for the Faces Pain Scale, compared to the Colored Analog Scale, among children in all age groups and for both genders [30]. 

The last part of the questionnaire focused on mandibular function impairment; the words “resistance with chewing” were replaced with “chewing at the extraction site” because the previous description was more related to TMD pain than to extraction pain. 

The psychometric properties of the Arabic version of the post-extraction pain experience questionnaire were satisfactory. The questionnaire had a total Cronbach’s alpha value of 0.83, indicating an excellent level of internal consistency/reliability. The minimum acceptable value of Cronbach alpha is 0.7. Moreover, values higher than 0.90 are considered too high and may indicate a redundancy of items rather than a homogeneity [6]. The original two studies of the questionnaire had Cronbach’s values that ranged from 0.63 to 0.95 across the different domains [12,13].

The questionnaire achieved a satisfactory level of content validity (Average CVI = 0.90). In total, 32 of 33 items on the questionnaire were considered “content valid,” and only 1 item scored less than 0.78 and was deleted. Careful selection of experts for reviewing the scale was made, based on their qualifications and experience, as well as their familiarity with the extraction procedure through clinical practice. These selection guidelines were recommended by Grant and Davis [31]. 

Spearman’s correlation showed a strong correlation between the scale we used in this study (Part I) and the VAS scale (local anesthesia r = 0.63, extraction r = 0.77, and the total score r = 0.75), indicating good criterion validity. However, the correlation with the SEM scale was weak (local anesthesia r = 0.36, extraction r = 0.48, and total score r = 0.36). Although both SEM and VAS are considered valid scales for assessing pain in children [24,32], VAS is a self-reporting scale, while SEM is a behavioral rating scale. The poor correlation with SEM was consistent with another study that found a lack of correlation between self-reporting measures and behavioral rating scales [33]. Behavioral rating measures can be misleading when used solely in pain assessment and result in under or overestimation of the pain intensity. Overestimation can result when anxiety is high during the procedure, and as a result, influences pain perception. Studies have also shown that high anxiety levels evoke a greater pain response [2]. However, some children may be able to control their behavior, which could result in low scoring on the behavioral rating scale, and thus, an underestimation of the pain intensity [2]. Poor correlation can also result because dental injections and extractions are anxiety evoking procedures; studies have also shown that it is not always possible to distinguish between anxiety and pain. They are thought to have considerable overlap in their physiological and psychological components [24]. It is, thus, recommended to use an anxiety scale, such as the Dental Anxiety Scale (DAS), in addition to a pain assessment for extraction procedures in children [34]. 

The main goal when using the factor analysis method is to obtain the optimal, easily interpretable factor structure. All variables should have a single high loading in one factor. This was achieved by re-running the analyses several times and making a few modifications. For example, any variables with cross-loadings, low communality values, and low factor loadings were deleted e. Moreover, the application of the orthogonal (Varimax) rotation technique simplified the factor structure and facilitated factor interpretation by making each factor independent of the other factors [15]. The resulting factor model had well-defined, highly interpreted factors. Based on the factor analysis, 15 items were divided into four factors. Factor 1 was labeled “analgesic consumption” and comprised four items. Factor 2 was labeled “expression of discomfort from the extraction site” and consisted of four items. Factor 3 was labeled “perception of masticatory ability” and consisted of 3 items. Factor 4 was labeled “pain/discomfort from the dental extraction procedure” and consisted of four items. The labeling of “factor 3” was in agreement with Stangenga et al. [12].

It was noted that factor loadings in items related to pain and discomfort from local anesthesia procedures seemed to be low compared to the other items in the factor model. This may be related to the wide variations of scoring obtained. Differences related to the technique of local anesthesia administration used (the use of topical anesthesia or the chasing technique vs. direct palatal infiltration, nerve block vs. infiltration), the clinician’s level of expertise (postgraduate vs. undergraduate students) were all possible explanations for differences in the obtained scores.

Items related to pain from the extraction site on the first evening and after one week were not included in the factor structure due to their low factor loading. As mentioned earlier, for our sample size, a factor loading of <0.5 was not considered significant. In addition, the children’s inability to differentiate between pain and discomfort was another possible explanation for low factor loading, most likely due to their young age (mean age = 9.9). One study showed that many dentists were of the opinion that children had difficulties in differentiating between pain and discomfort [3]. Moreover, other studies showed that the extraction of primary teeth caused a low level of pain perception, which explains the difficulty of reporting pain and discomfort separately [11,35]. The use of analgesics was also considered a plausible explanation for the low values of reported pain/discomfort on the first evening after extraction [11]. Future studies could combine the items of pain and discomfort from the extraction site on the first evening and after one week, as presented in the Dental Discomfort Questionnaire (DDQ), which focuses on recognizing toothache or discomfort in children [36]. 

Our study has some limitations, the main one being enrolling a convenience non-random sample. Dental services at King Abdulaziz University Dental Hospital are mostly offered to children of low socioeconomic status who may, due to psychological aspects or cognitive awareness levels, express their pain perception differently from children who are treated in private clinics. Therefore, caution should be taken when interpreting our results. Other variations were in the clinical settings, such as the type of primary teeth extracted and the level of the operators’ qualifications. These variations were overcome by using different validity methods, and the results were strengthened by not relying solely on a single test. Nevertheless, larger, more generalized, samples should be considered in future studies. Finally, it was not possible to assess the test-retest reliability because children cannot appropriately report pain on assessments that are taken two weeks apart. 

In summary, the questionnaire nevertheless had satisfactory reliability, and validity. The resulting shorter model, which consisted of 15 items, can indeed be used in the selected age group to assess pain and discomfort after the extraction of primary teeth. But further studies with a larger, more representative, sample are still to be recommended. 

## Figures and Tables

**Table 1:** A self-reported questionnaire comprising 33 items that assessed pain, discomfort, analgesic consumption, daily activities, and jaw-function impairment.

**1. Immediately after extraction**	**1** فورًا بعد عملية الخلع
1.1 Pain during injection	1.1 هل تألمت أثناء حقن مادة التخدير؟
1.2 Pain during extraction	1.2 هل تألمت أثناء عملية الخلع؟
1.3 Discomfort during injection	1.3 هل تضايقت أثناء حقن مادة التخدير؟
1.4 Discomfort during extraction	1.4 هل تضايقت أثناء عملية الخلع؟
1.5 Was any part of the extraction particularly unpleasant?	1. 5 هل وجدت أي جزء من عملية الخلع كريه بشكل خاص؟
1.6 If so, explain which part was particularly unpleasant	1.6 إن أجبت بنعم، فما هو الجزء الذي كرهته بشكل خاص؟
**2. First evening after extraction**	**2** المساء الأول بعد عملية الخلع
2.1 Pain from extraction site *	2.1 هل عندك ألم من مكان الخلع الآن ؟
2.2 Discomfort from extraction site *	2.2 هل مكان الخلع يضايقك الآن ؟
2.3 Analgesic consumption *	2.3 هل تناولت مسكنات للألم؟
2.4 Kind of analgesics *	2.4إن أجبت بنعم، فما هو نوع مسكنات الألم التي استخدمتها ؟
**3. One week after extraction**	**3** بعد أسبوع من عملية الخلع
3.1 Pain from extraction site *	3.1هل عندك ألم من مكان الخلع الآن ؟
3.2 Discomfort from extraction site *	3.2هل مكان الخلع يضايقك الآن ؟
3.3 Analgesic consumption last week *	3.3هل تناولت مسكنات للألم خلال الأسبوع الماضي؟
3.4 Number of days using analgesics	3.4إن أجبت بنعم، فكم عدد الأيام التي تناولت فيها مسكنات الألم؟
3.5 Use of non-prescription drugs	3.5هل كان الدواء بدون وصفة طبية؟
3.6 Kind of analgesic *	3.6 إن أجبت بنعم، فماهونوع مسكن الألم الذي استخدمته؟
3.7 Use of prescription drugs	3.7هل كان الدواء بوصفة طبية؟
3.8 Kind of analgesic	3.8 إن أجبت بنعم، فماهونوع مسكن الألم الذي استخدمته؟
3.9 School absence	3.9هل تغيبت عن المدرسة الأسبوع الماضي بسبب الألم في مكان الخلع؟
3.10 Number of days	3.10 إن أجبت بنعم، فكم عدد الأيام التي تغيبت فيها عن المدرسة؟
3.11 Leisure activities refrain	3.11 هل امتنعت عن القيام بالأنشطة الترفيهية الأسبوع الماضي بسبب الألم في مكان الخلع؟
3.12 Number of days	3.12 إن أجبت بنعم، فما هي الأنشطة التي امتنعت عن القيام بها؟
3.13 Sleep disturbance	3.13 هل اضطرب نومك في الأسبوع الماضي بسبب الألم في مكان الخلع؟
**4. Jaw-function impairment after one week**	**4** الخلل الوظيفي للفك إذا كانت لديك صعوبات بعد الخلع، فإلى أي مدى كان تأثيرها على كل مما يلي؟
4.1 Leisure	4.1 وقت فراغك
4.2 Speech	4.2 كلامك
4.3 To take a big bite	4.3 تناول لقمة كبيرة
4.4 To chew hard food	4.4 مضغ الطعام القاسي
4.5 To chew soft food	4.5 مضغ الطعام الليّن
4.6 School work	4.6 الواجبات المدرسية
4.7 To drink	4.7 الشرب
4.8 To laugh	4.8 الضحك
4.9 To chew on extraction site	4.9 مضغ الطعام مكان الخلع
4.10 To yawn	4.10 التثاؤب

* Four repeated measurements collected at two-time points; from the first evening after extraction and from the first week after extraction. The table mentioned the items in both Arabic and English languages.

**Table 2:** The SEM scale [24] used to measure pain and discomfort.

Observations	1. Comfort	2. Mild Discomfort	3. Moderately Painful	4. Painful
Sounds	No sounds indicating pain	Nonspecific sounds; possibly indicating pain	Specific verbal complaints, e.g., “ow”, raising voice	Verbal complaints indicating intense pain, e.g., screaming, sobbing
Eyes	No eye signs of discomfort	Eyes wide, showing concern, but no tears	Watery eyes and/or flinching eyes	Crying, tears running down face
Motor	Hands relaxed; no apparent body tenseness	Hands show some distress or tension, grasping chair due to discomfort, muscular tension	Random movements of arms or body without aggressive intention to make physical contact, grimace, twitch	Movements of hands trying to make aggressive physical contact, e.g., punching, pulling head away

**Table 3:** Criterion validity analysis of questionnaire pain and discomfort scores vs. SEM and VAS.

	vs. SEM	vs. VAS
**Questionnaire** **Local Anesthesia** **Scores**	r = 0.36	r = 0.63
	(*p* = 0.0097)	(*p* < 0.0001)
	weak correlation	strong correlation
**Questionnaire** **Extraction Scores**	r = 0.48	r = 0.77
	(*p* = 0.0004)	(*p* < 0.0001)
	moderate correlation	strong correlation
**Total Score**	r = 0.36	r = 0.75
	(*p* = 0.01)	(*p* < 0.0001)
	weak correlation	strong correlation

Statistically significant *p* < 0.05 using Spearman’s rho correlation. SEM (sound, eye, motor scale), VAS (visual analog scale).

**Table 4:** Content validity analysis; ratings of 33-item scale by Five experts.

Items	Expert 1	Expert 2	Expert 3	Expert 4	Expert 5	Number in Agreement	Item CVI
**Questions immediately after extraction**							
1.1	X	X	X	X	X	5	1
1.2	X	X	X	X	X	5	1
1.3	X	X	X	X	-	4	0.8
1.4	X	X	X	X	X	5	1
1.5	X	-	-	X	X	3	0.6
1.6	X	X	X	X	X	5	1
**Questions first evening after extraction**							
2.1	X	X	X	X	X	5	1
2.2	X	X	X	X	X	5	1
2.3	X	-	X	X	X	4	0.8
2.4	X	X	X	X	X	5	1
**Questions one week after extraction**							
3.1	X	-	X	X	X	4	0.8
3.2	X	-	X	X	X	4	0.8
3.3	X	-	X	X	X	4	0.8
3.4	X	X	X	X	-	4	0.8
3.5	X	X	-	X	X	4	0.8
3.6	X	X	X	X	X	5	1
3.7	X	X	-	X	X	4	0.8
3.8	X	X	-	X	X	4	0.8
3.9	X	-	X	X	X	4	0.8
3.10	X	X	X	X	X	5	1
3.11	X	X	X	X	-	4	0.8
3.12	X	X	X	X	X	5	1
3.13	X	X	X	X	X	5	1
**Functional Jaw impairment after 1 week**							
4.1	X	X	-	X	X	4	0.8
4.2	X	X	X	X	X	5	1
4.3	X	X	X	X	X	5	1
4.4	X	X	X	X	X	5	1
4.5	X	X	X	X	X	5	1
4.6	X	X	X	X	-	4	0.8
4.7	X	X	X	X	X	5	1
4.8	X	X	X	X	X	5	1
4.9	X	X	X	X	X	5	1
4.10	X	X	X	X	X	5	1
**Proportion relevant**	1	0.81	0.84	1	0.87	Mean I-CVI = 0.90 Mean expert proportion = 0.90	

I-CVI (item-content validity index); X indicates item rated 3 or 4 on a 4-point scale; Mean expert proportion is the average proportion of items judged relevant across the five experts = 0.90.

**Table 5:** The factor structure after Varimax orthogonal rotation.

15 items with factor loading >0.5	**Factor 1**	**Factor 2**	**Factor 3**	**Factor 4**
3.3 Analgesics (after 1 week)	**0.97601**	0.08083	0.04242	−0.03450
3.4 Number of days taking analgesics (after 1 week)	**0.94949**	0.04214	0.14526	0.03189
3.5 Nonprescription drugs (after 1 week)	**0.97539**	0.08983	0.05328	−0.01819
3.6 Kind of analgesics (after 1 week)	**0.88750**	0.23602	−0.04188	0.05994
3.2 Discomfort from extraction site (after 1 week)	0.01886	**0.67665**	0.13159	0.00716
4.1 Leisure	0.12581	**0.85896**	0.04080	0.06984
4.2 Speech	0.18339	**0.66459**	0.39500	0.12720
4.6 School work	0.14400	**0.85807**	−0.01563	−0.13424
4.4 To chew hard food	−0.00090	0.21960	**0.80151**	0.06150
4.5 To chew soft food	0.12383	−0.12429	**0.77466**	−0.04103
4.9 To chew on extraction site	0.00057	0.43592	**0.75448**	−0.02718
1.1 Pain with local anesthesia	−0.13831	0.00163	−0.04434	**0.58476**
1.2 Pain with extraction	0.04441	0.22440	−0.06731	**0.78819**
1.3 Discomfort with LA	0.08652	−0.12778	−0.01373	**0.53109**
1.4 Discomfort with extraction	0.04587	0.00012	0.16412	**0.79893**
Eigen Value	4.3768	2.6250	1.9073	1.5497
% of explained variance	29.18	17.50	12.72	10.33

The highest factor loadings are highlighted in bold. Factor 1: Analgesic consumption. Factor 2: Expression of discomfort from extraction site. Factor 3: Perception of masticatory ability. Factor 4: Pain/discomfort from the dental extraction procedure.

## References

[1] Merskey H.A.F.D., Bonica J.J., Carmon A., Dubner R., Kerr F.W.L., Lindblom U., Mumford J.M., Nathan P.W., Noordenbos W., Pagni C.A. (1979). Pain terms: A list with definitions and notes on usage. Recommended by the IASP Subcommittee on Taxonomy. Pain.

[2] Young K.D. (2005). Pediatric procedural pain. Ann. Emerg. Med..

[3] Wondimu B., Dahllöf G. (2005). Attitudes of Swedish dentists to pain and pain management during dental treatment of children and adolescents. Eur. J. Paediatr. Dent..

[4] Hla T.K., Hegarty M.K., Russell P., Drake-Brockman T.F., Ramgolam A., Von Ungern-Sternberg B.S. (2014). Perception of Pediatric Pain: A comparison of postoperative pain assessments between child, parent, nurse, and independent observer. Pediatric Anesth..

[5] Kimberlin C.L., Winterstein A.G. (2008). Validity and reliability of measurement instruments used in research. Am. J. Health Pharm..

[6] Streiner D.L., Norman G.R., Cairney J. (2015). Health Measurement Scales: A Practical Guide to Their Development and Use.

[7] Merkel S.I., Voepel-Lewis T., Shayevitz J.R., Malviya S. (1997). The FLACC: A behavioral scale for scoring postoperative pain in young children. Pediatric Nurs..

[8] Downie W.W., Leatham P.A., Rhind V.M., Wright V., Branco J.A., Anderson J.A. (1978). Studies with pain rating scales. Ann. Rheum. Dis..

[9] Garra G., Singer A.J., Taira B.R., Chohan J., Cardoz H., Chisena E., Thode H.C. (2010). Validation of the Wong-Baker FACES Pain Rating Scale in Pediatric Emergency Department Patients. Acad. Emerg. Med..

[10] McGrath P.A., Seifert C.E., Speechley K.N., Booth J.C., Stitt L., Gibson M.C. (1996). A new analogue scale for assessing children’s pain: An initial validation study. Pain.

[11] Naoumova J., Kjellberg H., Mohlin B., Kurol J. (2011). Pain, discomfort, and use of analgesics following the extraction of primary canines in children with palatally displaced canines. Int. J. Paediatr. Dent..

[12] Stegenga B., De Bont L.G., De Leeuw R., Boering G. (1993). Assessment of mandibular function impairment associated with temporomandibular joint osteoarthrosis and internal derangement. J. Orofac. Pain.

[13] Feldmann I., List T., John M.T., Bondemark L. (2007). Reliability of a Questionnaire Assessing Experiences of Adolescents in Orthodontic Treatment. Angle Orthod..

[14] Campbell C.M., Edwards R.R. (2012). Ethnic differences in pain and pain management. Pain Manag..

[15] Hair J., Black W., Babin B. (2014). Multivariate Data Analysis.

[16] Beaton D.E., Bombardier C., Guillemin F., Ferraz M.B. (2000). Guidelines for the Process of Cross-Cultural Adaptation of Self-Report Measures. Spine.

[17] Hilton C.E. (2015). The importance of pretesting questionnaires: A field research example of cognitive pretesting the Exercise referral Quality of Life Scale (ER-QLS). Int. J. Soc. Res. Methodol..

[18] Nunnally J.C. (1978). Psychometric Theory.

[19] Streiner D.L. (2003). Starting at the Beginning: An Introduction to Coefficient Alpha and Internal Consistency. J. Personal. Assess..

[20] Waltz C.F., Strickland O.L., Lenz E.R. (2010). Measurement in Nursing and Health Research.

[21] Almanasreh E., Moles R.J., Chen T.F. (2019). Evaluation of methods used for estimating content validity. Res. Soc. Adm. Pharm..

[22] Lynn M.R. (1986). Determination and Quantification of Content Validity. Nurs. Res..

[23] Polit D.F., Beck C.T., Owen S.V. (2007). Is the CVI an acceptable indicator of content validity? Appraisal and recommendations. Res. Nurs. Health.

[24] Wright G.Z., Weinberger S.J., Marti R., Plotzke O. (1991). The effectiveness of infiltration anesthesia in the mandibular primary molar region. Pediatric Dent..

[25] Bailey B., Gravel J., Daoust R. (2012). Reliability of the visual analog scale in children with acute pain in the emergency department. Pain.

[26] Suhr D.D. Exploratory or confirmatory factor analysis? In Proceedings of the SAS Users Group International Conference (SUGI31), San Francisco, CA, USA, 26–29 March 2006.

[27] Williams B., Onsman A., Brown T. (2010). Exploratory factor analysis: A five-step guide for novices. Australas. J. Paramed..

[28] Maccallum R.C., Widaman K.F., Preacher K.J., Hong S. (2001). Sample Size in Factor Analysis: The Role of Model Error. Multivar. Behav. Res..

[29] Hogarty K.Y., Hines C.V., Kromrey J.D., Ferron J.M., Mumford K.R. (2005). The Quality of Factor Solutions in Exploratory Factor Analysis: The Influence of Sample Size, Communality, and Overdetermination. Educ. Psychol. Meas..

[30] De Tovar C., Von Baeyer C.L., Wood C., Alibeu J.-P., Houfani M., Arvieux C. (2010). Postoperative Self-Report of Pain in Children: Interscale Agreement, Response to Analgesic, and Preference for a Faces Scale and a Visual Analogue Scale. Pain Res. Manag..

[31] Grant J.S., Davis L.L. (1997). Selection and use of content experts for instrument development. Res. Nurs. Health.

[32] Bijur P.E., Silver W., Gallagher E.J. (2001). Reliability of the Visual Analog Scale for Measurement of Acute Pain. Acad. Emerg. Med..

[33] Beyer J.E., McGrath P., Berde C.B. (1990). Discordance between self-report and behavioral pain measures in children aged 3–7 years after surgery. J. Pain Symptom Manag..

[34] Corah N.L. (1969). Development of a Dental Anxiety Scale. J. Dent. Res..

[35] Sjögren A., Arnrup K., Jensen C., Knutsson I., Huggare J. (2010). Pain and fear in connection to orthodontic extractions of deciduous canines. Int. J. Paediatr. Dent..

[36] Versloot J., Veerkamp J.S.J., Hoogstraten J. (2006). Dental Discomfort Questionnaire: Assessment of dental discomfort and/or pain in very young children. Community Dent. Oral Epidemiol..

